# Supplementary material for: Plasmodium falciparum transcription in different clinical presentations of malaria associates with circulation time of infected erythrocytes
Source: Nat Commun. 2021 Jul 30;12:4711. doi: 10.1038/s41467-021-25062-z (PMC8324851; doi:10.1038/s41467-021-25062-z)
Supplement: Supplementary file 1 — Supplementary Information File [file 41467_2021_25062_MOESM1_ESM.pdf]

Supplementary Information File

P. falciparum transcription in different clinical presentations of malaria associates with circulation time of infected erythrocytes

Richard Thomson-Luque[1], Lasse Votborg-Novél[1,2], Wanangwa Ndovie[3], Carolina M. Andrade[1], Moussa Niangaly[2,4], Charalampos Attipa[3,5], Nathalia F Lima[1], Drissa Coulibaly[4], Didier Doumtabe[4], Bouréima Guindo[4], Bourama Tangara[4], Fayçal Maiga[4], Abdoulaye Kassoum Kone[4], Karim Traore[4], Kassoum Kayentao[4], Aissata Ongoiba[4], Safiatou Doumbo[4], Mahamadou A. Thera[4], Boubacar Traoré[4], Karl Seydel[6,7], Nuno S. Osório[8], Silvia Portugal[1,2*]

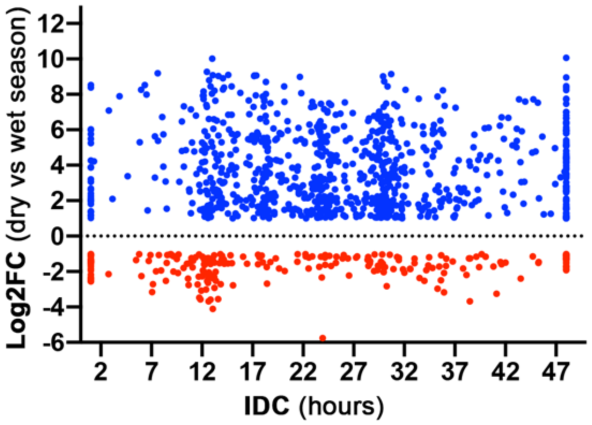

Supplementary Fig. 1 | Log_2 expression reported by Andrade et al. of 568 (50.22% of the total 1131) DEGs upregulated in low parasitaemias at the end of the dry season (blue), and 216 (45.38% of the total 476) DEGs upregulated in higher parasitaemias in malaria cases in the wet season (red), shown along the *X* axis according to Bozdech et al. peak timing of transcription.

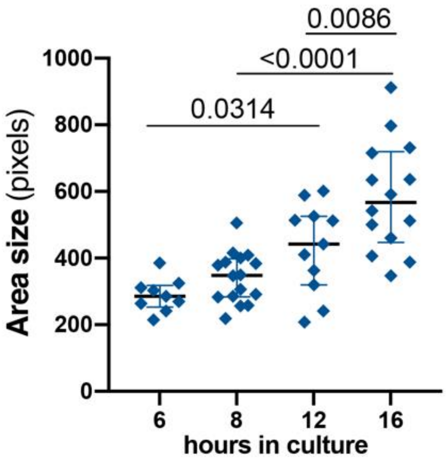

Supplementary Fig. 2 | 3D7 *P. falciparum* area measured from Giemsa-stained thick smears after 6, 8, 12 and 16 h in culture following merozoite invasion (n= 9, 16, 11 and 14 parasite measured respectively) . Data indicate median ± IQR; one-way ANOVA.
